# Organoids: technology refining, current applications and future directions

**DOI:** 10.1186/s43556-026-00422-7

**Published:** 2026-03-14

**Authors:** Xianda Cheng, Ziqi Fang, Jianhui Sun, Liyu Liu, Yan Yang, Junyi Wang, Jianwei Shuai, Xikun Zhou, Ping Lin, Gen Yang, Xiuli Bi, Min Wu

**Affiliations:** 1 College of Life Science, Liaoning University, Shenyang, 110036 China; 2 Wenzhou Institute, University of Chinese Academy of Sciences, Wenzhou, 325000 China; 3 School of Medical Information Engineering, Gannan Medical University, Ganzhou, 341000 China; 4 Biological Science Research Center, Southwest University, Chongqing, China; 5 Medical Research Center, The Third People’s Hospital of Chengdu, The Affiliated Hospital of Southwest Jiaotong University, Chengdu, 610031 China; 6 Laboratory of Allergy and Precision Medicine, Department of Respiratory Medicine, the Third People’s Hospital of Chengdu, Chengdu, China; 7 Department of Biotherapy, Cancer Center & State Key Laboratory of Biotherapy, West China Hospital, Sichuan University, and Tianfu Jincheng Laboratory Chengdu, Chengdu, 610041 China; 8 State Key Laboratory of Nuclear Physics and Technology, School of Physics, Peking University, Beijing, 100871 China

**Keywords:** Organoids, Immune organoids, Tumor, Immunotherapy, Tumor microenvironment

## Abstract

Organoids are derived from pluripotent stem cells or tissue stem cells, progenitor cells, or differentiated cells from healthy or diseased tissues (e.g., tumors). Numerous organoid engineering strategies have been tested to support the culture, growth, proliferation, differentiation, and maturation of organoids. A variety of organoids and organoid-on-chips have also been constructed to reflect real environments of human and mouse organs. Currently, four major areas of potential application for organoids include disease modeling, anticancer drug screening, drug toxicology testing, and gene/cell therapy. For cancer immunotherapy, immune organoids based on co-culturing human tumor cells have been used as a critical platform for drug screening and targeted therapy. This review summarizes recent advances in organoid culture, lists the methods for constructing organoids and their main applications, and highlights its value as a tool for precise cancer modeling. Given the enormous potential of organoids as an in vitro culture model in cancer treatment, we also discussed organoid-based methods for angiogenesis and immune microenvironment modeling, and analyzed the wide range of applications of immune organoids, such as testing treatment response, exploring mechanisms of drug resistance, optimizing treatment strategies, and guiding drug development. Finally, we attempt to look into the critical challenges and bright prospects for cancer organoid research.

## Introduction

Organoids are miniature cell clusters cultured and grown in a three-dimensional (3D) environment in vitro. Under the influence of cytokines, small molecule chemical inhibitors/activators, culture media, and other additives, these cell clusters self-organize and differentiate into functionally specific cell populations with tissue architecture and genetic characteristics similar to those of the corresponding organs [1]. Organoids offer advantages over traditional 2D cultures, displaying near-physiological cellular composition and behavior. Many organoids can be extensively expanded in culture and maintain genomic stability, making them suitable for biobanking and high-throughput screening [1, 2]. Compared to animal models, organoids reduce experimental complexity, are amenable to real-time imaging techniques, and enable the study of various aspects of human development and disease. From the brain, intestine, kidney, to pancreas, retina, and heart, a growing number of functionally mature organoids are emerging, providing new platforms for cancer research [3], drug screening [4], regenerative medicine [5], and even neuro-immunological studies [6].

Organoids are typically induced from pluripotent stem cells (iPSCs), stem cells from adult or fetal tissue, progenitor cells, or even differentiated cells. These cells are enzymatically hydrolyzed, fused with Matrigel, and then stimulated under various physical and chemical conditions [1]. Organoids can facilitate the development of precision medicine and personalized treatment—from simulating the tumor immune microenvironment and assessing immunotherapy responses to building disease models using CRISPR gene editing technology [7]. By simulating tumor microenvironment (TME), organoids provide a platform for evaluating the effects of immunotherapy drugs. Studies have shown that the antibody–drug conjugate Cet-ZA can activate Vd2 T cells and exert anti-tumor effects [8]; and the combination of PD-1 inhibitors and platinum-based compounds can enhance T cell secretion of IFN-γ and activate immune pathways [9]. In addition, organoid models are used to study STING pathway activation [10], AURKA inhibitors [11], and off-target toxicity of T cell-binding bispecific antibodies [12], providing a theoretical basis for discovering new therapeutic targets and optimizing treatment plans. Although tumor organoids, as a type of in vitro model, hold great potential for precision medicine and drug screening in cancer treatment, and mRNA tumor vaccines based on organoid screening have emerged in recent years, achieving some successes in specific patient populations (e.g., melanoma patients) [13], the therapeutic efficacy and prognostic prospects for most solid tumors remain limited. Many of these approaches fail to produce durable therapeutic effects. Tumor heterogeneity, therapeutic resistance, and the complexity of the TME continue to pose significant challenges in cancer research [14]. Furthermore, the complex and diverse immune mechanisms within the TME hinder the development of novel immunotherapies. A core strategy in current immunotherapy research is to alter the role of immunosuppressive immune cells within the TME to restore immune surveillance and cytotoxic activity [15]. Developing personalized immunotherapy approaches tailored to the extent and nature of immune cell infiltration within distinct TMEs could significantly improve clinical outcomes and cost-effectiveness [16].

Currently, many studies are dedicated to integrating human immune cells into tumor organoids, thereby generating "immune organoids" that can partially or completely reproduce human immune functions in vitro [17]. Although the concept of immune organoids is still being refined, the successful integration of immune cells into tumor organoids makes it possible to accurately simulate key immune processes in the tumor environment. This is a landmark development, given that most tumor organoids lack intrinsic immune cells. Therefore, immune organoids provide a promising platform for studying tumor-immune interactions and advancing immuno-oncology research [18]. This essay introduces the core steps and main applications of organoid construction, as well as the great potential of immune organoids in tumor treatment and their limitations. This article focuses on organoid technology, a cutting-edge biological model system, and systematically reviews its overall development trajectory and broad application prospects, from its general construction strategies and application scenarios. Critically, the article analyzes the inherent limitations of current tumor organoid models in simulating the tumor microenvironment, particularly immune responses, which constitute a critical bottleneck in existing research paradigms. To address the insurmountable challenge, we focus on the emerging paradigm of "immune organoids," emphasizing their immense potential in accurately simulating tumor-immune interactions, revolutionizing immunotherapy strategy evaluation, and promoting the development of personalized therapies by reconstructing functional immune system components. This is also a core innovation and critical theme of this article. Finally, we discuss the future development of organoid technology, especially integrated immune organoid models, in basic research and clinical translation. This review aims to clarify the urgency and necessity of developing immune organoid technology through systematic analyses, providing crucial theoretical perspectives to help overcome the shortcomings of current tumor research models and usher breakthroughs in immunotherapy.

## Basics of organoid technology

Stem cells play a crucial role in maintaining the normal structure and function of human organs, and their behavior is regulated by the surrounding microenvironment [19, 20]. To mimic this environment in experiments, scientists have developed numerous methods, such as using hydrogels and microfluidic devices, to precisely control the interactions between cells and the chemical and physical signals they receive. As early as 1977, researchers extracted a matrix called Matrigel from mouse tumors to support three-dimensional cell growth, laying the early foundation for the study of organoid [1]. Organoids are three-dimensional miniature tissues formed by stem cells self-organizing in vitro, which can mimic the function and structure of real organs to a certain extent. Compared to traditional flat cell culture and animal experiments, organoids are more closely aligned with the characteristics of the human body and are easier to manipulate experimentally [20, 21]. Therefore, they are widely used in drug development, personalized medicine, and disease mechanism research. However, organoids are not perfect. For example, they may lack a rich variety of cell types, have unstable structural development, and often lack important components, such as vascular and immune systems [21]. To improve the stability and function of organoids, researchers are seeking to further control the development of stem cells by leveraging knowledge from developmental biology [22].

### Source of organoid generation

**Fig. 1:**
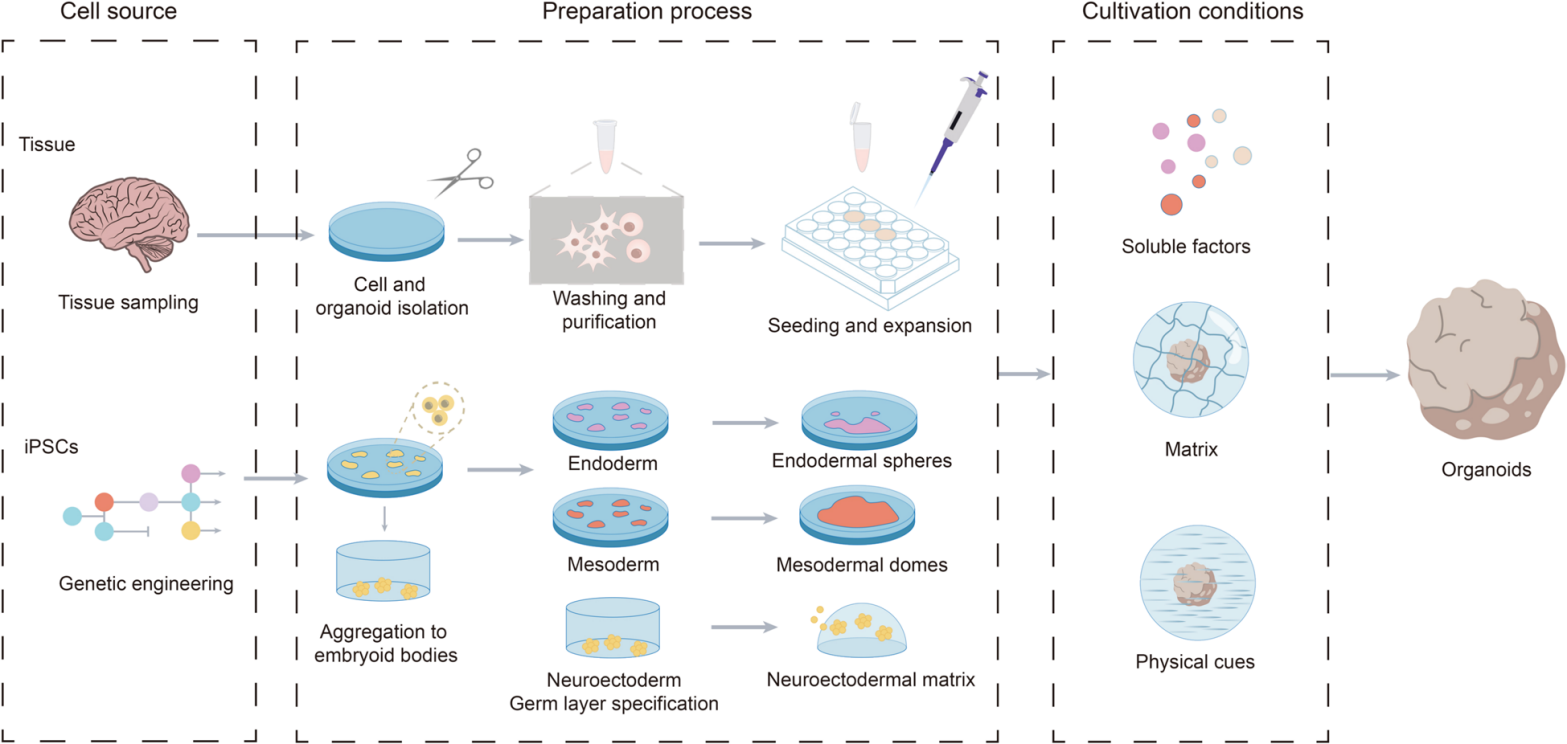
Organoid construction process. Organoids can be generated from tissue-derived cells (TDCs) or induced pluripotent stem cells (iPSCs). To generate organoids from TDCs, tissue samples are obtained from humans or animals (e.g., the intestine and stomach). The intestinal tissue sample is opened, washed, and cut into small fragments (2–4 mm) to increase the surface area for enzymatic digestion or further mechanical dissociation to isolate individual intestinal stem cells or crypts. After several rounds of washing and purification, the harvested stem cells or crypts are used to seed and generate organoid cultures for expansion

When establishing organoids, the starting cell source is crucial, affecting not only the stability of the resulting tissue structure but also its function similarity to a real organ. Currently, organoids of many organs can be generated from induced pluripotent stem cells (iPSCs), stem cells from adult or fetal tissues, progenitor cells, and even differentiated cells [21, 23, 24]. Different cell sources require distinct processing methods (Fig. 1). To establish organoids from tissue samples, the tissue must first be broken down to extract stem cells or tumor cells. This is typically accomplished through enzymatic or mechanical methods [25, 26]. Enzymatic digestion is the most common method, but the specific enzymes used, the combination of enzymes, and the duration of the digestion process vary depending on the tissue type. Other enzymes may also be added to remove DNA released by necrotic cells. Mechanical methods are more feasible, simpler, faster and more economical, but cell viability may not be as stable as enzymatic digestion. Often, both methods are used in combination. Intestinal organoids are first cut into small segments and chemically treated to remove intercellular connections. Stem cells from the intestinal crypts are then extracted and implanted in a three-dimensional Matrigel-like matrix, allowing them to naturally develop into organoids [27].

For iPSCs, cell lines are typically established and validated before being expanded in large quantities under specific conditions. These cells have poor survival in vitro, and are typically cultured in clusters to maintain intercellular contact and improve survival. Gently scraping procedures can also be used to manipulate irregular clusters during in vitro culture [28].

Tumor cells can be extracted from surgical tissue, puncture biopsies or body fluids. Some tumor cells are small in number and need to be expanded in animals (xenotransplantation) before being used for organoids [29–31]. Tumor tissue often contains normal cells at the same time, so it is difficult to completely separate them before culture. To solve this problem, the characteristic of tumor cells with a reduced dependence on certain nutritional factors can be used to design selective culture media, making it difficult for normal cells to grow, while tumor cells can be expanded [32]. In addition, red blood cell contamination in the sample may also affect organoid growth and matrix stability, so it is usually necessary to use lysis methods to remove blood cells substrates [33].

### Establishing organoids using a general approach

After cell isolation, it is common to embed the cells in a natural matrix like Matrigel, or in a natural extracellular matrix (ECM) material like collagen, or in synthetic hydrogels. Matrigel's composition is similar to that of basement membranes, rich in proteins like laminin, type IV collagen, and proteoglycans [34, 35]. When culturing intestinal organoids, intestinal crypt cells are first suspended in ice-cold Matrigel and then dripped onto a warm culture plate, forming a hemispherical cell–matrix structure. Culture medium is then added to allow the cells to grow [36, 37].

In organoid culture, researchers typically perform regular "passaging", re-fractionating grown organoids into smaller units and re-implanting them into new matrices for continued culture. Proliferation is also assessed by counting. Although Matrigel is widely used, its complex composition and instability, coupled with a lack of controllability, have led to the use of alternative materials with defined compositions, such as human recombinant collagen, fibrin, or various synthetic hydrogels [38, 39]. Synthetic hydrogels can individually tailor their physical and chemical properties, such as stiffness, viscoelasticity, and cell adhesion signals, to precisely influence the morphology and function of organoids [40]. Dynamic PEG-based hydrogels can be tailored to support stem cell growth or induce differentiation, or to precisely release space through photodegradable structures [41]. Furthermore, some hydrogels can support organoid growth for extended periods, even enabling them to develop intestinal-like crypt structures [42]. In addition, some hydrogels can support the growth of organoids for a long time and even enable them to form structures similar to renal tubules [43]. In addition to embedding cells directly into the matrix, cells can also be aggregated into clusters before implantation to form the "starting point" of organoids. The simplest method for clustering is to use ultra-low-attachment culture dishes, which prevent cells from attaching to the dish bottom and encourage them to adhere. Centrifugation can accelerate this process. Alternatively, the size and shape of each cluster can be controlled using microplates, or cells can be aggregated at the bottom of a droplet using the hanging drop method [44].

### Physical and chemical environmental control

Organoid culture is based on our understanding of developmental biology, specifically how cells in the body receive signals from their surroundings at specific times and locations [45]. To mimic this process in vitro, researchers typically add various soluble factors, such as growth factors (proteins) or small molecules, to the culture medium, which control the direction of cell differentiation [45, 46].

These factors help guide cells from their initial state (adult stem cells or iPS cells) into cells of a specific tissue type [47]. Protein-based growth factors are expensive and unstable, whereas small molecules, despite cheaper, can have broad effects, leading to poor reproducibility. Therefore, many protocols combine these two. To reduce costs, some experiments use conditioned medium: engineered cells secrete the desired growth factors, such as Wnt factors, and then use these cells' culture medium to culture organoids [48, 49]. However, this approach can also be inconsistent between batches, leading to the development of alternative molecules to improve stability. In a real physiological environment, cells are not exposed to these signals randomly; rather, these factors are released in a precisely controlled manner by neighboring cells or the matrix [50]. Therefore, when and where these signals are added during organoid culture is crucial. This spatiotemporal regulation is especially crucial when inducing iPSCs to form complex organs [51]. Adjusting the timing of adding different signaling factors can better mimic in vivo developmental processes. To achieve this precise regulation, researchers have developed a variety of techniques. Growth factors can be encapsulated in nanoparticles or directly attached to the cell surface for slow or controlled release. Alternatively, growth factors can be immobilized using heparin-containing materials [45], mimicking the in vivo ECM, allowing them to interact with cells in a more natural manner [52].

Other advanced nanotechnologies, such as the use of nanostructures (nanopillars [53], nanopits [54], and nanochannels), create an environment more similar to the natural basement membrane, enabling organoids to grow more effectively in three dimensions [55]. Microfluidic chip systems can also be used to simulate microscopic physiological environments, precisely controlling the distribution of nutrients, gases, or signaling factors. A neural tube microfluidic device has been developed that can create opposing growth factor gradients within organoids, mimicking the natural development of the nervous system [56].

In addition to chemical signals, physical factors are also crucial for organoid growth. As organoids grow, their cellular demand for nutrients increases, but the diffusion efficiency of nutrients and metabolic waste decrease, easily leading to hypoxia and necrosis in the central region. This is particularly evident in intestinal and brain organoids. If brain organoids are too large, the central region is prone to death due to insufficient nutrient supply [57, 58]. To address this issue, researchers have adopted various approaches, including regular fragmentation and re-culturing [59]. Intestinal organoids are often cut into small pieces and replanted to ensure adequate nutrient penetration. The use of agitated culture systems, such as shaking cultures and rotating bioreactors, allows for better nutrient flow and remove waste; and the use of microfluidic chips (miniature culture systems) continuously supplies nutrients to organoids and removes metabolic waste, promoting long-term and stable growth [60].

In addition to nutrient supply, the surface structure of the material also influences cell behavior. Cells undergo changes in morphology and arrangement when placed on surfaces with varying uneven textures. These physical signals can also influence the direction of stem cell differentiation. When culturing intestinal organoids on specific soft materials, the surface structure of the material can influence the organoid's growth morphology [61].

In short, to make organoids as similar as possible to real organs, it is important not only to provide them with the right signals but also to maintain them in the growing-friendly environment—including flow, temperature, oxygen, and even the structure of the culture dish surface.

### Signal integration

While organoid culture initially relied on the self-organizing ability of cells, researchers now prefer to integrate multiple physiologically relevant signals to more precisely control organoid growth and morphological development. For intestinal organoids, researchers first isolated LGR-labeled stem cells from intestinal tissue, seeded them in Matrigel, a substrate that mimics the natural basement membrane, and added appropriate growth factors. This resulted in the successful growth of most stem cells into organoids. However, this approach still suffers from significant individual variability and structural instability, and organoids can vary in size, shape, and function [62]. To address this, scientists have combined physical cues (such as substrate stiffness) with chemical cues (such as growth factors). They found that gradually softening the culture substrate from stiff to soft can better guide organoid maturation. Similar approaches have also been applied to the culture of neural organoids, using micropatterning techniques to mimic the mechanical and chemical gradients within tissue, successfully inducing the morphological development of the neural tube [63, 64]. In addition to microenvironmental manipulation, more advanced engineering technologies have also been introduced into organoid research like 3D bioprinting, which uses bio-ink to precisely print cells into specific structures that mimic the structure of real organs [65]. Organ-on-a-chip as miniature devices incorporated microfluidic channels enables simultaneous cultivation of multiple organoids and mimic the blood flow and inter-organ communication found in the body [66]. Collectively, by integrating multiple signals and engineering approaches, scientists are gradually overcoming the structural instability and imperfect function of organoids, bringing them closer to real organs. This has important implications for disease modeling and drug development [67].

### Atlas of organoid types

The organoids currently being researched and discussed can essentially be categorized into two types: regenerative medicine organoids and tumor organoids [68], which differ in their sources and application areas. Regenerative medicine organoids can be further divided into epithelial organoids, multi-tissue organoids, and multi-organ organoids [69] (Table 1). Regenerative medicine organoids are artificial tissues created through biomedical engineering methods and cell culture techniques to mimic the structure and function of natural organs [70]. These tissues can mimic the structure and function of natural organs but are typically cultured in the laboratory, which are composed of multiple cell types, forming functional "micro-organs". These cells are crucial because they can better simulate the developmental processes and physiological and pathological states of organ tissues [71]. They can be used in regenerative medicine, developmental biology, gene function, material bioactivity, drug toxicity and sensitivity [70, 71]. In particular, stem cell technology can be used to culture patient-matched organs, addressing donor shortages and avoiding immune rejection [72].

**Table 1:** Classification of organoids

Organoid class atlas			
Category	**Organoids for regenerative medicine**		**Tumor organoids**
Source	Pluripotent stem cells (PSCs) include: embryonic stem cells (ESCs), induced pluripotent stem cells (iPSCs)	Adult stem cells (ASCs): a source of healthy tissue	Tumor tissue or malignant effusions such as pleural effusion and ascites
Features	They have the ability to self-renew indefinitely and differentiate into nearly all organ-specific cell types	It can differentiate into various cell types that are highly consistent with the tissue, has the ability to self-assemble, and the tissue structure is highly biomimetic to the cell type; it generally only contains the epithelial part of the organ and lacks the matrix, nervous and vascular systems	It highly maintains tumor heterogeneity and heterogeneity between patients, and has the advantages of genome stability after multiple passages and short culture cycle
Type	Liver organoids, lung organoids, kidney organoids, brain organoids, etc	Digestive system organoids (liver, pancreas, colorectum, stomach, etc.), respiratory system organoids (alveoli, bronchial tubes), reproductive system organoids (ovaries, cervical endometrium, vaginal epithelium, etc.)	Breast cancer, colorectal cancer, lung cancer, prostate cancer, esophageal cancer, pancreatic cancer, etc
Application	Regenerative medicine, developmental biology, gene function, material bioactivity, drug toxicity and sensitivity, and other fields		Disease modeling, research and drug screening, personalized precision medicine and other fields for cancer patients

Epithelial organoids are the most widely studied type of organoid and their structures are derived from a single germ layer (endoderm, mesoderm, or ectoderm) that possesses the ability to self-renew under appropriate culture conditions [73]. In this context, self-renewal describes the repeated regeneration of organoids from organoid fragments or single cells, allowing for the continuous expansion of cultures [73]. Epithelial organoids demonstrate this characteristic through the ability of these structures to form from the clonal expansion of single cells. As epithelial organoids expand, cells polarize and specialize to replicate aspects of the native epithelium [74]. As the name suggests, epithelial organoids lack the mesodermal components typically present in native tissues. In some cases, epithelial organoids are co-cultured with supporting cells; however, these cells may not turn to part of the epithelial organoid. Furthermore, in order to fully represent/simulate an specific organoid, the cells must be functionally integrated into the overall structure and synchronized with the organoid's proliferative state [75].

Multitissue organoids are established by co-culturing cells from at least two germ layers or by co-differentiation of PSCs [76]. In the liver, pancreas, and biliary tract, multitissue organoids are composed of cells of both endodermal and mesodermal origin. Unlike epithelial organoids, current protocols do not support self-renewal of multitissue organoids, requiring a coordinated expansion of parenchymal and supporting cell types [77]. Instead, cells interact to achieve stable levels of maturity and function. One advantage of multitissue organoids is their tissue-like heterocellular composition. Multitissue organoid systems are well-suited for studying heterotypic cell–cell interactions among multiple cell types commonly found in native tissues. Importantly, these cultures demonstrate intra-organ self-organization between epithelial and supporting cell types, similar to native tissues [78].

Multi-organ organoids are the most complex and least described type of organoids, which is characterized by self-organized patterns of organoid development. These systems hold great promise for studying organogenesis, a process controlled by the interactions of multiple tissue boundaries [79].

Tumor organoids are three-dimensional structures derived from patient tissue obtained through biopsy, aspiration, or surgical resection, cultured in Matrigel for several weeks [80]. They are an important tool for studying tumor pathology and pathogenesis, as they maintain a high degree of heterogeneity within the original tumor and between patients while preserving nearly uniform morphology and dimensions across individuals [81, 82]. They provide a rapid and robust platform for studying tumor pathogenesis, drug screening, and personalized precision medicine. Using culturing patient tumor tissue, personalized tumor organoids can be established to assess individual responses to different drugs and select the most appropriate treatment options [83].

## Massively popular applications of organoids

**Fig. 2:**
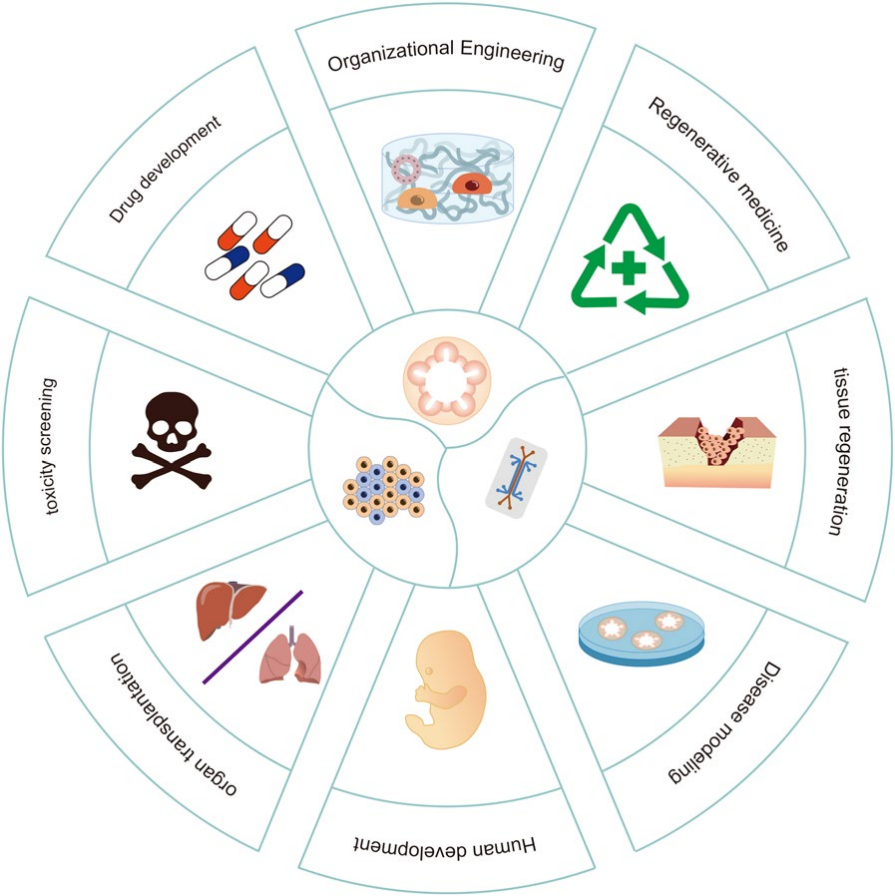
The applications of organoids. As an emerging in vitro culture platform, organoids are widely used in drug screening, toxicology assessment, tumor treatment and regenerative medicine

Since researchers successfully induced the culture of intestinal organoids from Lgr5 stem cells, organoids of varying needs have been rapidly utilized. Since organoids can closely mimic the genetic and epigenetic characteristics of target tissues or organs, they hold broad application prospects in various areas including organ development, precision medicine, regenerative medicine, drug screening, gene editing, and disease modeling (Fig. 2) [84].

### Organogenesis and regenerative medicine

Organ transplantation remains the only treatment option for end-stage diseases of vital organs like the heart, liver, kidneys, and lungs, yet only approximately 10% of global organ demand are met annually. Regenerative medicine is a highly promising treatment approach aimed at repairing or replacing damaged organ tissue with healthy, functional cells [85, 86]. Organoids, a revolutionary technology of the twenty-first century, are simulating the development and disease mechanisms of human organs with unprecedented precision, providing an ideal research model for regenerative medicine. Organoid models, like miniature organs, can form three-dimensional tissue structures with specific organ functions in vitro [87]. Furthermore, through the combination of cell engineering techniques, organoids can be expanded in large quantities, providing an excellent cell source for translational applications [88]. Therefore, the emergence of organoid technology has opened up new horizons for regenerative medicine research and brought new hope to humanity in overcoming the challenges of tissue repair and organ transplantation [89].

Patient-derived organoids enable the use of (autologous) cells for tissue-compatible organoid regeneration and cell therapy without the risk of alloreactivity or rejection. Organoids hold great potential in regenerative medicine, particularly autologous organoid transplantation, which minimizes the risk of immune rejection [90]. Indeed, the feasibility of transplantation, in vivo functional engraftment, and regeneration of liver [91], colon [37, 92], and pancreas organoids [93] have been demonstrated in various mouse models. For example, Kruitwagen et al. transplanted autologous gene-corrected canine liver organoid cells into COMMD1-deficient dogs (this transplantation strategy can also be extended to human clinical trials), and evaluated the engraftment, reimplantation and functional recovery of liver disease. They also reported the long-term survival of these cells (up to 2 years) in canine models related to hereditary copper poisoning (e.g., Wilson's disease) [94]. In recent years, research on regenerative medicine and organoids is not limited to traditional organoid transplantation, but also includes the optimization of regenerative medicine organoid culture systems [95] and the use of organoid models to explore specific genetic mechanisms [95, 96].

### Disease modeling

Organoid technology is emerging as an application enabling us to begin to understand diverse aspects of a wide range of diseases [97], such as microcephaly [98], autism [99], ulcerative colitis [100], and Crohn's disease [101]. Gene-editing technologies (e.g., CRISPR/Cas9) offer the potential to be applied to organoids derived from patients with single-gene defects, such as Huntington's disease [102] and polycystic kidney disease (PKD) [103], to generate healthy tissue for transplantation. Currently, disease modeling around organoids has developed into co-culture systems of organoids and cells (smooth muscle cells) [104, 105], or viral infection models developed for viral infection [104, 105]. Furthermore, while many challenges and hurdles remain, the establishment of a biobank of patient-derived organoids will further enhance our understanding of inter- and intra-patient heterogeneity and potentially enable personalized treatments for a variety of diseases [106].

### Drug screening and toxicological evaluation

Organoid technology plays a key role in the development and evaluation of new drugs. While organoids are not directly involved in new drug development, they are crucial for identifying promising drug candidates through drug screening and testing [107]. Organoid cultures are used for drug screening and can also correlate the genetic background of tumors with drug response [108]. In liver cancer research, the establishment of organoids from healthy tissue of the same patient provides an opportunity to develop less toxic drugs by screening compounds that selectively kill tumor cells without harming healthy cells. Hepatocyte organoid cultures with self-renewal capacity can be used to test potential new drugs for liver toxicity (one of the reasons for drug failure in clinical trials) [109]. Overall, organoid technology has advanced all stages of drug development, providing extensive assistance throughout the entire process and helping to optimize drug development costs and improve efficiency [110]. Using organoids for drug screening offers the following advantages: short turnaround time: high organoid construction success rates and rapid culture speed. Typically, drug screening can be performed after one week of organoid culture. The entire process, from sample collection to drug susceptibility results, can now be well controlled within two weeks. In terms of the drug throughput screening, organoids can not only screen multiple drugs on the well plate, but also test each drug at different concentrations, and multiple experiments can be carried out in parallel [111, 112].

Organoid technology offers multiple advantages, such as low cost, short development time, and greater physiological relevance. Organoids, obtained through extensive expansion in in vitro culture systems while maintaining stable physiological and genetic information over long periods of time, are closer to clinical practice [113]. Organoid models can increase the success rate of screening for drugs with greater physiological and clinical relevance, accelerate the identification of drugs that will benefit patients, and accelerate drug clinical application [114]. Compared to other models, organoids redefine the landscape of biomedical models, offering a more physiologically relevant alternative. Addressing challenges of standardization, scalability, physiological relevance, and ethical considerations will be key to fully leveraging organoids in toxicology research [115]. With continuous technological advancements and interdisciplinary collaboration, organoid models offer the opportunity to revolutionize toxicology, leading to more accurate and efficient testing of hazardous substances, significantly improving human health and safety [116]. Organ-on-a-chips can reflect the dynamic behavior of drugs in vivo and the authentic responses of human organs to drug stimulation, addressing the significant deviations from existing models [117]. They constitute a comprehensive drugability evaluation system encompassing pharmacokinetic, pharmacodynamic, and toxicological properties, enabling researchers to intuitively assess the safety and efficacy of new drugs [118].

## Current challenges: simulating the tumor microenvironment

Although in vitro organoids have broad applications in disease modeling and pathological and toxicological validation, their use in cancer treatment is still limited due to a number of uncertainty. The main limitation lies in the difficulty of replicating the complex microenvironment and intercellular interactions with non-tumor cells that occur during tumor development in vivo, which makes tumor organoids unsuitable for developing emerging immunotherapies and personalized medicine. On the other hand, a number of good features of immuno-organoids have transformed them to a promising alternative.

### The complexity of the tumor immune microenvironment

**Fig. 3:**
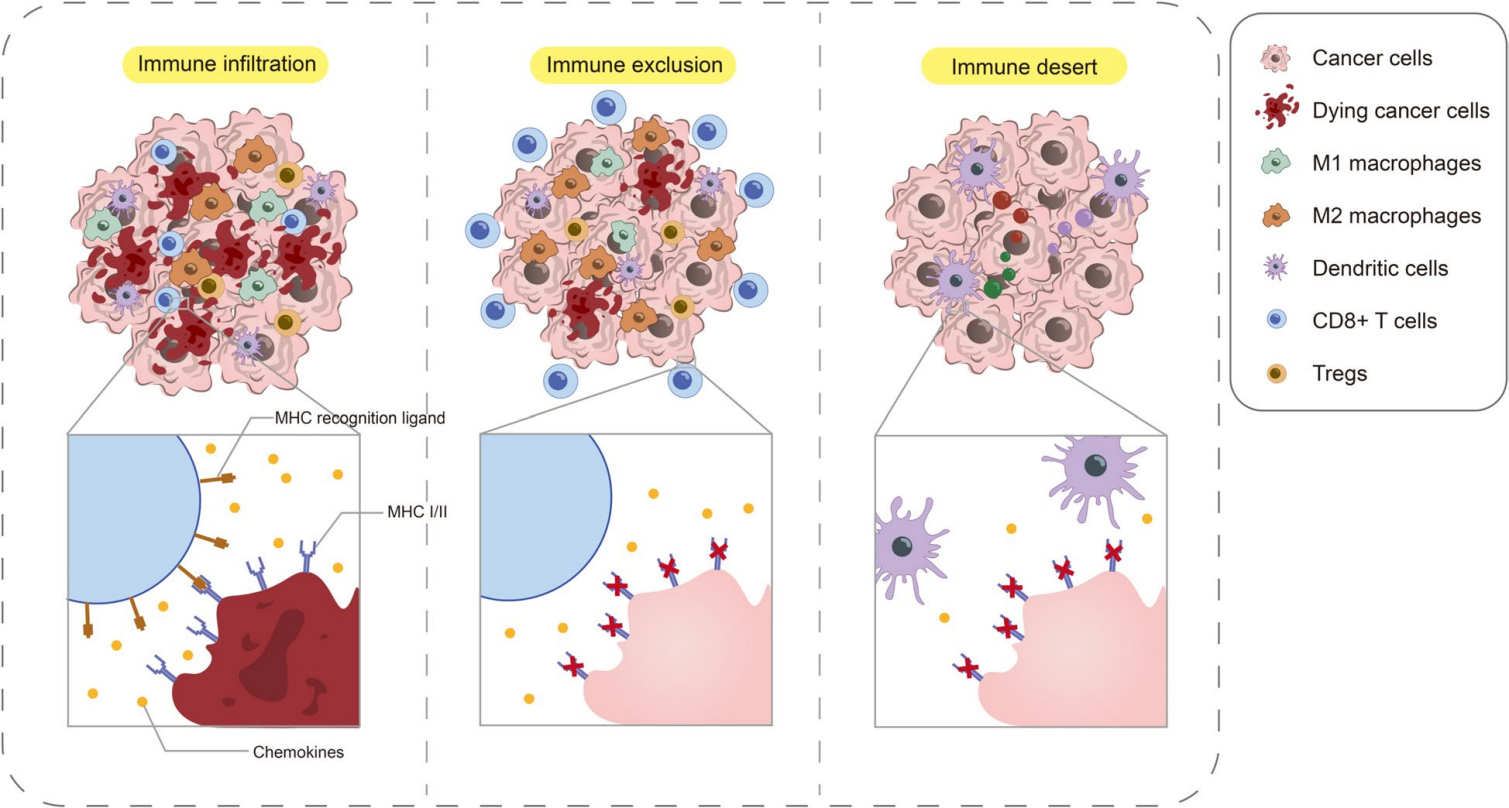
Tumor immune microenvironment classification. The tumor immune microenvironment is commonly categorized into three types: immune infiltration, immune rejection, and immune desert. The immune infiltration type is characterized by abundant infiltration of CD8⁺ T cells and M1 macrophages, which can effectively recognize and eliminate tumor cells. The immune rejection type exhibits more significant immunosuppression due to the presence of M2 macrophages, Th cells, and Treg cells. This subtype also shows reduced antigen recognition and increased immune evasion. The immune desert type is mainly devoid of immune cell infiltration

Initially, the use of the human immune system to combat tumors was met with skepticism. This was partly due to its delayed therapeutic effects compared to the immediate impact of surgery or chemotherapy and partly due to an incomplete understanding of the complex human immune system at the time [119]. However, as immune checkpoint inhibitors entered large-scale clinical trials for various cancers, a major challenge became evident: while some patients responded favorably to immune checkpoint inhibitors, others did not respond at all [120, 121]. This inconsistency prompted investigations into changes within the TME across different tumor types [122]. Recent advances in understanding tumor–immune interactions have led to the classification of tumors based on their immune phenotype. Tumors were initially categorized into "hot" tumors characterized by lymphocyte infiltration and inflammation and "cold" tumors without lymphocyte infiltration and inflammatory responses in the TME [123]. Further subclassification divided hot tumors into immune-rejection and immune-infiltration phenotypes, while cold tumors were refined as immune-desert types (Fig. 3) [124, 125].

Immune-infiltrated tumors, also known as inflammatory tumors [126], are characterized by the infiltration of a large number of immune cells, such as macrophages, Treg cells, Th cells, and NK cells in the tumor microenvironment [127]. Compared to immune-rejection tumors, CD8⁺ T cells in immune-infiltration tumors can penetrate the tumor tissue and exert cytotoxic effects. Immune-infiltration tumors often exhibit high expression of MHC molecules, which increases immune surveillance and promotes tumor cell eradication [128]. Immune-infiltration tumors demonstrate the most favorable responses to immune checkpoint inhibitor therapy [129]. On the other hand, immune-rejection tumors present a more complex scenario [130]. Their microenvironment contains immunosuppressive cells that protect tumor cells from immune detection [131]. Although CD8⁺ T cells are frequently present at the invasive margin, their inability to infiltrate the tumor is a hallmark of immune-excluded phenotypes, with limited responsiveness to immune checkpoint inhibitors [132]. Immune-desert tumors are devoid of immune cell infiltration and are characterized by impaired antigen presentation (low MHC I expression) and aggressive tumor growth [133, 134]. These tumors often exhibit low tumor mutational burden (TMB), genomic stability, and poor responses to monotherapy with immune checkpoint inhibitors [135]. Furthermore, rapidly proliferating immune-desert tumors frequently undergo metabolic reprogramming that further alters the TME, and fibrosis of the tumor extracellular matrix induces mechanical reprogramming of TAMs, which are already present at low levels, thereby creating an immunosuppressive environment [136, 137].

**Fig. 4:**
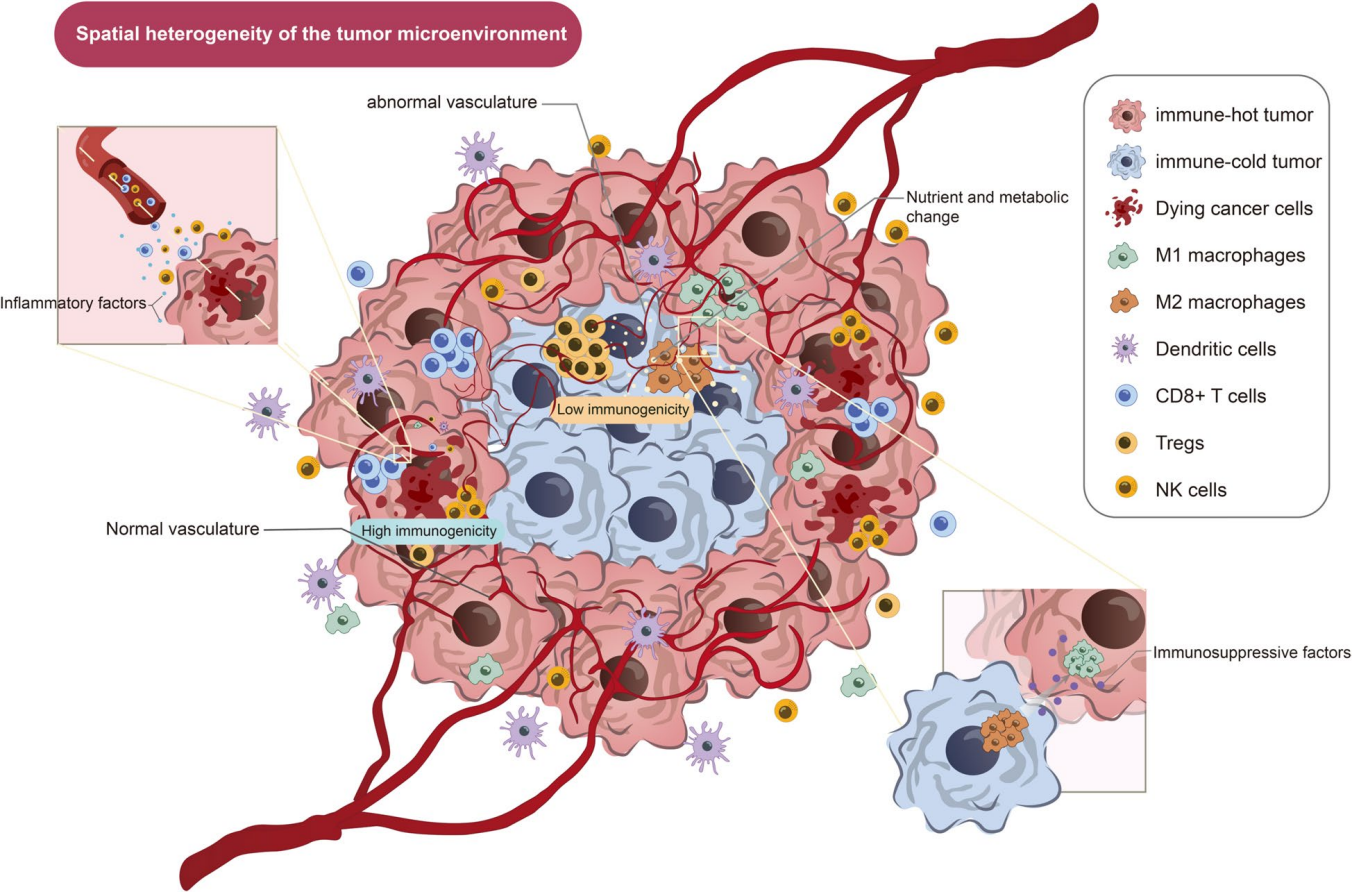
Spatial heterogeneity of TME. The immune microenvironment varies spatially within tumor regions due to uneven tumor cell distribution. At the tumor margin or near normal vasculature, immune cell accumulation leads to improved antigen recognition, facilitating tumor cell elimination. On the other hand, the tumor core, influenced by angiogenesis and metabolic alterations, often develops an immunosuppressive environment suitable for tumor growth. Within this region, the functions of M1 macrophages and CD8⁺ T cells are suppressed by M2 macrophages and Treg cells, resulting in immune escape

During carcinogenesis and metastasis, malignant cells become increasingly heterogeneous (Fig. 4) [138], which allows tumors to recruit various immune-related elements, including cytokines and chemokines [139, 140]. Most solid tumors exhibit immunological heterogeneity, which evolves both temporally and spatially due to tumor progression and therapeutic pressure. The variability of anti-tumor immunity is associated with the course of the illness and its response to treatments [141]. The characteristics of the tumor immune microenvironment are largely determined by tumor and non-tumor components (immune cells, extracellular matrix, and immune factors) [142], including immune checkpoint molecule expression, secretion of immunosuppressive mediators, vascular structure, proximity to tumor margins, and availability of metabolic resources [143]. These spatial differences within the TME can significantly affect clinical prognosis and therapeutic response [144].

RNA sequencing analysis in patients with pancreatic ductal adenocarcinoma revealed significant differences in immune cell composition [145], which was reflected in lower infiltration of CD8⁺ T cells and dendritic cells, along with increased accumulation of immunosuppressive cells [146]. As tumors progress, the continuous accumulation of these immunosuppressive cells will cause the tumor immunosuppressive microenvironment to continue to be maintained, and the proportion of immune cells that perform killing functions will gradually decrease until they no longer exist, which is more conducive to tumor development and angiogenesis [147]. Importantly, the presence of immunologically adverse regions or lesions within a single patient is associated with poor clinical outcomes, further underscoring the significance of spatial and temporal heterogeneity in disease progression and treatment response [148, 149].

### Challenges for tumor organoids research in cancer therapy

In recent years, organoid models of patient-specific tumors are changing the understanding of cancer heterogeneity and the impact of personalized medicine. These advances are largely due to the ability of organoid models to stably preserve the genetic, proteomic, morphological and drug characteristics of parent tumors in vitro, and provide new opportunities for genomic and environmental manipulation. However, current cancer organoid culture technology has problems of uncontrollability and non-reproducibility [150]. Although researchers have tried to retain the tumor microenvironment as much as possible in organoid models, key components such as immune cells and fibroblasts in the tumor microenvironment will still gradually disappear during the continuous culture of organoids, making the difference between organoids and parent tumors gradually increase, making it difficult to truly reflect the tumor microenvironment and accurately predict the effect of immunotherapy. Studies have found that the patient-derived tumor fragment (PDTF) model containing primary tumor cells and original microenvironment components directly obtained from patients can predict the efficacy of immune checkpoint inhibitors with an accuracy of more than 73%. However, the culture method of amplifying organoids in vitro and then adding microenvironmental components to restore the characteristics of the parental tumor microenvironment cannot accurately predict the efficacy of immune checkpoint inhibitors [151, 152]. In addition, the occurrence and development of tumors may be regulated by multiple organ systems. Nevertheless, it is currently difficult to establish a culture system in which tumors coexist with other tissues and organs in vitro, and it is also difficult to conduct basic research on the interaction between the body's macroenvironment and the local microenvironment of tumors. Optimizing the in vitro culture platform to more effectively simulate the growth environment of tumors in vivo is also another important direction for tumor organoid research [153].

### Merits of immune organoids in tumor treatment

Recent fast progress in immunotherapy makes this area an emerging field in cancer treatment. By engineering a patient's immune cells, researchers could improve the tumor microenvironment while minimizing immune rejection, activating relevant immune cells to kill tumors. However, research in the field of cancer treatment and emerging immunotherapies have gradually exposed the limitations of traditional tumor organoids. Their relatively simple cell composition and small number of immune cells make it difficult to explore tumor-infiltrating immune cells and corresponding immunotherapies. Therefore, immune organoids designed to be compatible with immunotherapies have emerged. Given the complex composition of the tumor microenvironment (TME) during tumor development, there is an urgent need for advanced in vitro models that are different from two-dimensional culture and traditional tumor organoid culture to restore the complex characteristics of the TME. Immune organoid technology has emerged as a result [154]. Immune organoids are considered to be a new model for studying human biology and disease etiology because they have a spatial organization similar to that of the corresponding organs, retain some key features, useful for studying the development and interaction of immune cells, such as T cell maturation or immune response. Immune organoids can restore the complex immune microenvironment in vivo to a certain extent [155]. Compared with traditional tumor organoid models, immune organoids primarily consist of immune cells (T cells, B cells, dendritic cells, macrophages, etc.) and may contain supporting matrix cells (such as fibroblasts) [156]. This means that immune organoids have the advantages of in vitro models similar to tumor organoids, since both immune cells and tumor cells originate from the patient, which can highly retain the epigenetic characteristics of patients, and provide certain guidance and suggestions for personalized medicine for patients through drug screening, toxicity testing, etc. In addition, immune cells regularly extracted from patients’ peripheral blood or lymphatic organs can be directly or indirectly co-cultured with tumor organoids. By utilizing the interaction between immune cells and various cells including tumor cells, the immune infiltration microenvironment of "cold tumor" or "hot tumor" in the tumor immune microenvironment can be restored [157, 158], thereby achieving a certain degree of reproduction of the complex tumor microenvironment in vivo, while also avoiding the defect of traditional tumor organoid culture that the non-tumor components become less and less [159–161]. By applying different stimuli to immune organoids, the temporal and spatial heterogeneity of the tumor immune microenvironment can also be observed. These advantages are difficult to achieve with ordinary two-dimensional culture and basic tumor organoid culture [162]. The observation of the reproduction, heterogeneity and interaction of immune cells in the tumor immune microenvironment can be used to explore the interaction mechanism between the immune cells and TME in the preclinical stage, and in clinical translational research, it can be used to develop immunotherapy (CAR-T, checkpoint inhibitors), and evaluate vaccine effects, or infection immune models (simulating HIV infection of CD4^+^T cells) (Table 2) [163–165].

**Table 2:** Differences between immune organoids and tumor organoids

	**Tumor organoids**	**Immune organoids**
Core goals	Simulating tumor development	Simulate the immune system, focusing on the development and interactions of immune cells
Main cell types	Tumor cells, a few epithelial cells	Tumor cells, tumor-infiltrating immune cells, epithelial cells
Cultivation methods	Requires stimulation with epithelial/tumor growth factors (EGF, FGF, WNT, etc.)	Requires immune cytokine stimulation (IL-2, IL-7, SCF, etc.)
Application scenario	Explore cancer progression, drug screening, mutation analysis, and personalized treatment	Study antibody production, T/B cell interactions, immune activation, vaccine efficacy, and T/B cell function
Technical challenges	Maintaining tumor heterogeneity and complexity	Maintaining and restoring the complexity of the tumor immune microenvironment

## Construction and culture of immune organoids

**Fig. 5:**
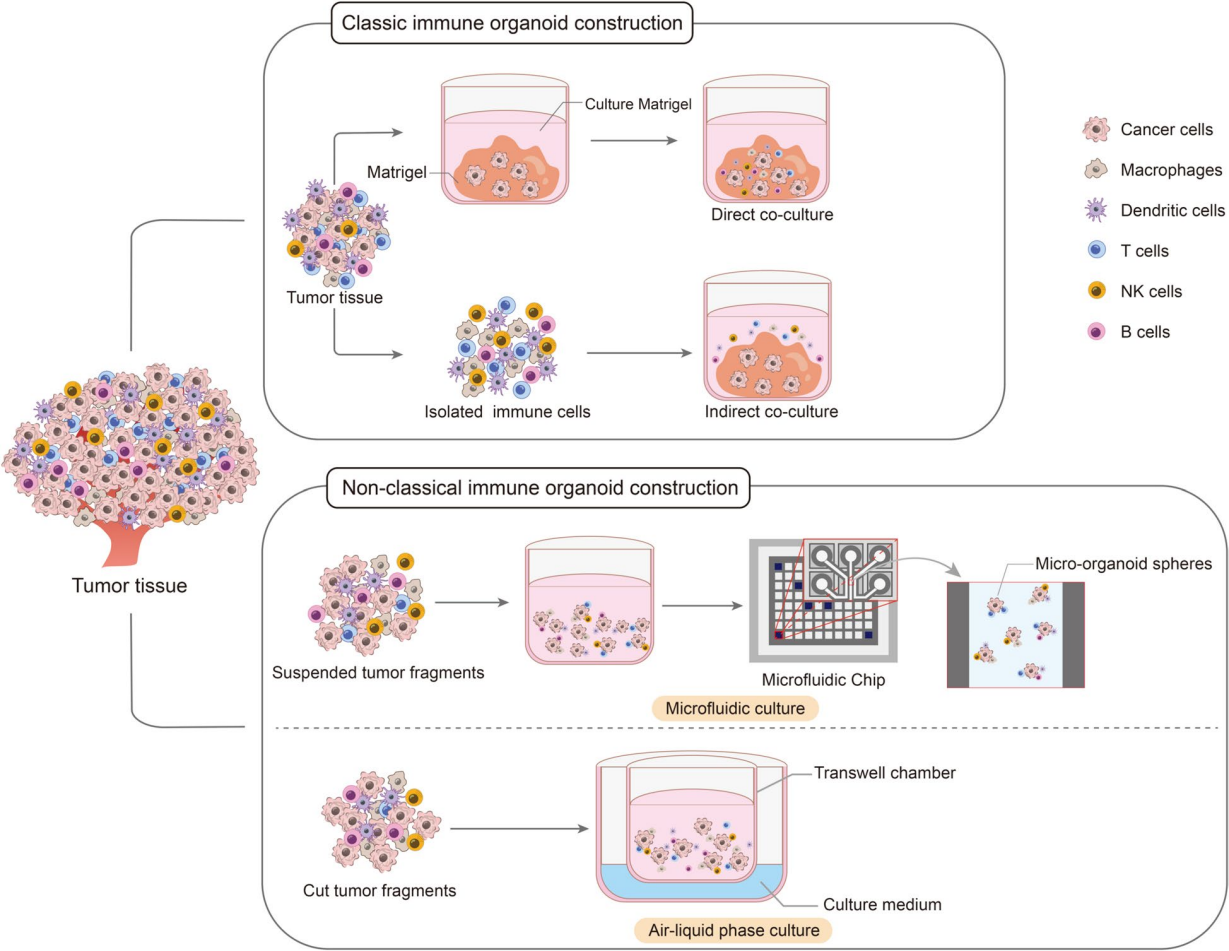
Construction methods of immune organoids. Immune organoids are typically constructed using a matrix gel immersion culture. Technological advances have allowed the development of alternative approaches, including air–liquid interface culture and microfluidic culture systems

The integration of tumor organoids with immune cells allows the development of 3D models that closely resemble the in vivo TME, offering a valuable system for precision cancer research and therapy. As a rapidly advancing research platform, tumor immune organoids recapitulate critical features of the TME, therefore facilitating the investigation of tumor initiation, progression, and immune interactions [166]. Currently, immune organoids are primarily established using three main culture approaches: immersion matrix gel culture, air–liquid interface (ALI) culture, and microfluidic culture (Fig. 5) [157]. Each method presents unique advantages in preserving tissue architecture, maintaining immune cell viability, and enabling long-term culture, thus supporting a wide range of tumor immunology applications.

### Immersion matrix gel culture

Immersion matrix gel culture represents one of the earliest methods developed for organoid generation. This was also one of the earliest methods for constructing immune-related organs [167, 168]. Tumor organoids derived from patient tumors can accurately replicate the architectural and molecular features of their in vivo counterparts [169, 170]. Unlike traditional organoid matrix gel culture, immune organoids also require co-culturing immune cells with cells from different sources to maintain a specific immune microenvironment, aiming to conduct immune-related research under conditions that are closer to the physiological environment. Two main co-culture strategies are employed: (1) Creating tumor organoids first, followed by the co-culture of immune cells isolated from peripheral blood, and (2) Introducing immune cells directly during the organoid establishment phase [171].

A common and straightforward co-culture approach involves directly adding exogenous immune cells to Matrigel-embedded tumor organoids [172]. In this setting, immune cell-secreted cytokines can affect tumor cells, although direct cellular contact may be limited by the matrix. Immune cells with strong infiltrative ability may penetrate the gel and interact with tumor cells [173]. For instance, Xu et al. immersed melanoma organoids in Matrigel and introduced both CD8⁺ T cells and anti-PD-1 antibodies. They observed the reactivation of CD8⁺ T cells and tumor cell apoptosis. This treatment method, which activates the immune function of immune cells, reshapes the tumor microenvironment, and thus kills tumor cells, is consistent with the strategy of in vivo immunotherapy to inhibit tumor development. Therefore, this model has broad application prospects in the evaluation of immunotherapy [174]. Currently, there are methods for constructing organoids using collagen, extracellular matrix or synthetic hydrogels, but they are less used in the construction of immune organoids. If the subsequent construction of immune organoids can be separated from the matrix gel scaffold, it will greatly reduce the construction cost of immune organoids and provide better prospects for this field [174].

Although the indirect co-culture strategy has many advantages, the matrix can interfere with immune cell function, potentially inducing nonspecific activation. To study direct tumor–immune interactions more precisely, extracting organoids from the gel may be beneficial before combining them with immune cell suspensions [175]. Giselbrecht et al. directly stimulated intestinal organoids with macrophages. They found that intestinal tissue-specific responses varied depending on the physical distance between organoids and macrophages, which may retain the spatial heterogeneity of the tumor immune microenvironment to a certain extent [176]. Furthermore, Alizadeh et al. established patient-derived lymphoma organoids and demonstrated that therapeutic bispecific antibodies against lymphoma organoids induced B cell death and T cell activation [161]. It can be observed that, compared to indirectly co-cultured immune organoids, directly embedding immune cells and tumor cells in matrix gel can better simulate or truly represent the spatial heterogeneity of the immune microenvironment. However, due to the highly differentiated nature of immune cells, even if good culture conditions can be maintained, it is difficult to avoid the phenomenon that immune cells will gradually decrease over time. Compared to indirect co-culture systems that can replenish immune cells in a timely manner, direct co-culture may be more difficult to maintain for a longer culture time.

### Air–liquid interface (ALI) culture

Although ALI culture was one of the earliest methods used to construct basic organoids, its characteristic of direct exposure to air allows for convenient supplementation of immune-stimulating factors (such as IL-2) and provides sufficient oxygen supply. Therefore, this technique has been widely applied to the culture of immune organoids. The method involves mechanically dissecting immune-related organ tissues into small fragments and placing them in a Transwell culture dish with a permeable membrane. This dish is placed in a culture plate containing culture medium, so that the lower part of the dish is in contact with the medium, while the upper surface of the organoid is exposed to the air. This setup simulates the air–liquid interface in vivo, promoting cell differentiation and maintaining tissue structure [177]. Kuo et al. employed ALI culture to develop a human autoimmune organoid model for studying the pathogenesis of celiac disease (CeD) [178]. Using endoscopic biopsy samples, they generated duodenal organoids that retained native epithelium, stromal components, and tissue-resident immune cells, offering novel insights into CeD immunopathology [178]. Furthermore, they extended the application of ALI technology by generating organoids from murine gastrointestinal tissues, including the intestine, stomach, and pancreas, using mechanical dissociation and collagen-based ALI culture. This method was then adapted to surgically remove primary and metastatic human tumor specimens, yielding ALI patient-derived organoids (PDOs) from over 100 tumor samples across 19 tissue types and 28 distinct malignancies. These organoid samples retain the characteristics of tumor tissue and associated immune-infiltrating cells, and can restore the complex interaction system between tumor-infiltrating immune cells and cancer cells [178]. Unlike matrix gel immersion co-culture, which reshapes the immune microenvironment, ALI-cultured immune organoids are generated by culturing tumor fragments that have been enzymatically digested or physically dissected. These organoid models can maintain and expand endogenous immune cells and can essentially preserve the patient's natural tumor immune microenvironment. Therefore, they have a wider range of applications in drug screening or personalized medicine.

### Microfluidic culture

The integration of microfluidic chip technology with organoid systems has enabled the development of "organoid-on-a-chip" platforms, which improve physiological relevance by providing precise control over the microenvironment [162]. Microfluidic technology related to immune organoids is different from traditional organoids constructed through microfluidics. The patient's tumor tissue and immune cells are cut into individual "organoid balls", and then the organoids are combined with matrix materials through microfluidics technology, and then the mixture is injected into oil-containing droplets. These droplets can form spontaneously at the oil–water interface, and each droplet contains a combination of immune organoid balls and matrix materials. Microfluidic-based organoid culture systems improve nutrient exchange, spatial architecture, and dynamic interaction between tumor and immune components [179]. Therefore, similar to ALI culture, this is also an in vitro culture technique for constructing and culturing immune organoids that can preserve the patient's native immune microenvironment to the greatest extent. Shen et al. utilized droplet emulsion-based microfluidics with precise temperature regulation and minimal dead volume to generate large numbers of microscale organoids from low-volume patient tissues [180]. These "micro-organospheres" (MOS) can support rapid, high-throughput immuno-oncology drug screening. The platform facilitates the retention of native stromal cells and promotes T-cell infiltration, offering a promising tool for personalized immunotherapy testing in clinical oncology [180]. Similarly, Zhang et al. designed a multilayered microfluidic chip that allows co-culture of tumor organoids with immune cells, simulating immune infiltration in hepatocellular carcinoma [181]. This system allows high-throughput drug screening while maintaining spatial and functional integrity of immune-tumor interactions. The organoid-on-chip model demonstrates increased accuracy in simulating tumor immunobiology and holds the potential for evaluating immunotherapeutic efficacy in hepatocellular carcinoma and other solid tumors [180, 181].

## Application of immune organoids in tumor immunity research

**Fig. 6:**
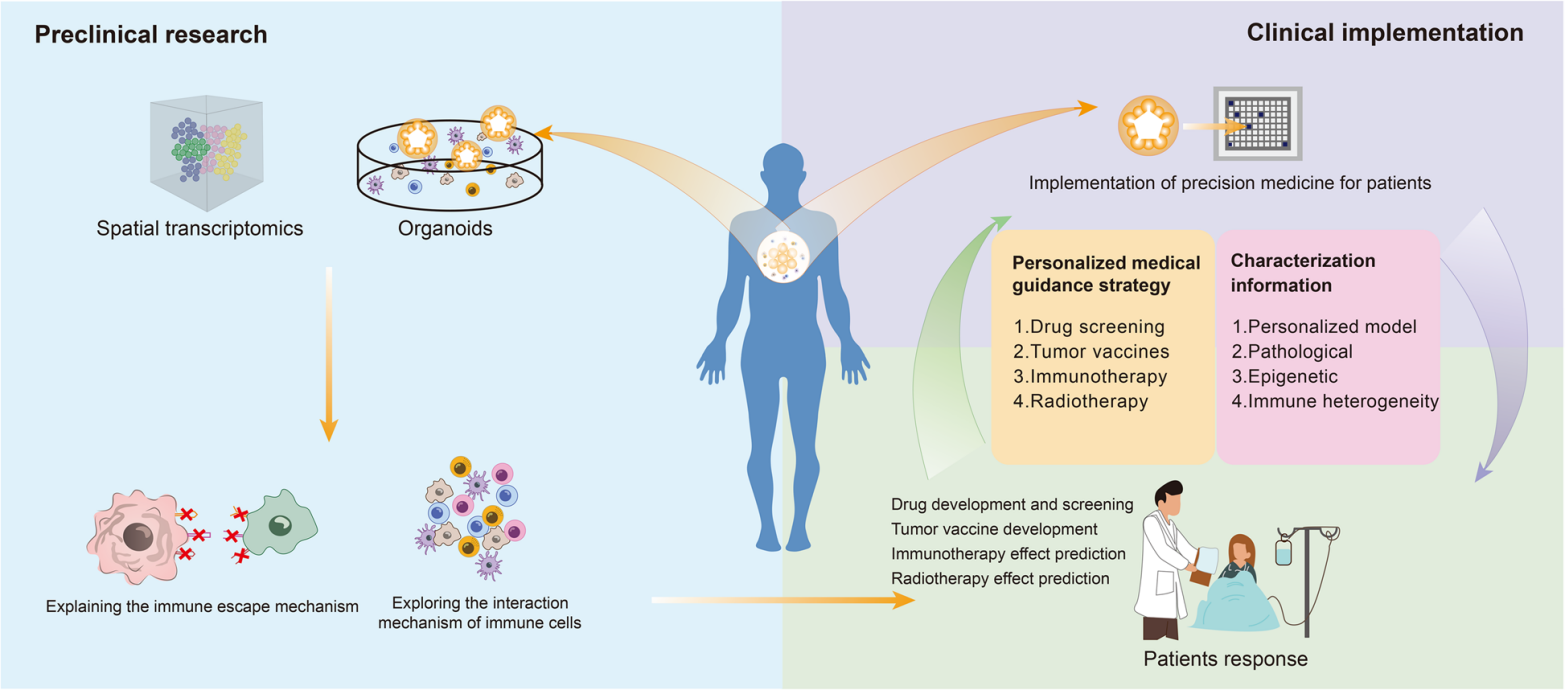
Applications of immune organoids. Immune organoids replicate both the reproducibility of in vitro systems and the complexity of the in vivo immune microenvironment. They are widely employed in drug screening, vaccine development, investigation of immune evasion mechanisms, precision medicine, and translational research, bridging laboratory findings with clinical applications

Immune organoids have been widely used in various fields of tumor research. Currently, research on immune organoids in tumor treatment chiefly contains tumor immune mechanisms, drug screening, predicting treatment response, and indirectly guiding individualized treatment strategies based on these aspects (Fig. 6) [182].

### Research on basic mechanisms with immune organoids

Advances in immune organoid systems have enabled us to explore the interactions between tumor cells and immune cells in more detail. This is crucial for elucidating the mechanisms of tumor initiation and progression [183]. Su et al. constructed immune organoids derived from gastric cancer and demonstrated that cancer-associated fibroblasts (CAFs) inhibited the cytotoxic function of natural killer (NK) cells. This immunosuppressive effect was mediated by CAF-induced NK cell ferroptosis [184]. These findings highlight the potential of immune organoids in simulating cell crosstalk in the tumor microenvironment (TME) and identifying immune regulatory mechanisms of matrix components.

ALI culture can often maintain the activity of patient-derived tumor cells and immune cells in vitro for 1–2 weeks. These in vitro models can often preserve the patient's epigenetic characteristics to the greatest extent, which makes it possible to combine immune organoid platforms with single-cell RNA sequencing to more thoroughly analyze the immune escape mechanisms of different types of TME tumors. Furthermore, the combination of spatial transcriptomics and immune organoids can better reveal the spatial heterogeneity of the patient's immune microenvironment. In addition to screening for specific immune cell clusters for further research, the spatial distribution characteristics of immune cells and tumor cells can also visually demonstrate the degree of tumor infiltration by immune cells during tumor development [185]. Lengner et al. used single-cell transcriptome profiles of colorectal cancer (CRC) to predict the interactions between cancer cells and TME components [186]. Subsequently, the researchers used immune organoids to simulate these interactions, thereby reproducing the dynamics of myeloid-cancer cell interactions in vitro. They found that tumor-like cultures suppressed gene expression programs associated with inflammation and immune cell migration, providing a simplified platform for reconstructing immune interactions in vitro [186].

### Immune organoids and preclinical translational platforms

As mentioned above, immune organoids isolated from patients not only serve as an important carrier for basic mechanism research, but also have a high degree of conservation of patient pathological characteristics, making them an important means of evaluating drug efficacy and toxicity, thereby accelerating the drug development process [187]. These models can simulate the transition of the tumor microenvironment (TME) from an immunosuppressive state to an immune-activated state after therapeutic intervention [188]. Liu et al. developed a gel-liquid interface (GLI) co-culture model combining lung cancer organoids (LCOs) with paired patient peripheral blood mononuclear cells (PBMCs) to simulate systemic anti-tumor immunity, optimizing cellular interactions via a superhydrophobic microporous chip [189]. This model can accurately predict the efficacy of immune checkpoint inhibitors (ICIs) in lung cancer patients. Its response index (Ri) is highly correlated with clinical outcomes and reveals that peripheral T cell infiltration and activation are key mechanisms of immunotherapy. This model, superior to conventional organoids or biomarkers, can dynamically track T cell differentiation trajectories and drug responses, laying a platform for personalized immunotherapy and revealing the immune synergy between peripheral T cells and tumor-draining lymph nodes [188–190].

In addition to the field of drug development, immune organoids also have great potential in the field of tumor vaccine development. Tumor vaccines are designed to activate the host immune system to fight cancer cells, which is a development trend in the field of tumor treatment. Immune organoids are a key platform for evaluating the design, immunogenicity, and efficacy of these vaccines [191]. Wang et al. developed an in situ dendritic cell vaccine, HELA-Exos, based on exosome engineering [192]. This approach significantly inhibited tumor growth in patient-derived immune organoids and in a poorly immunogenic triple-negative breast cancer (TNBC) xenograft model for the deadly subtype of breast cancer study [192].

### Immune organoids and engineered immunotherapy

Adoptive cell transfer therapy (ACT) is an innovative cancer treatment method that involves isolating immune cells from the patient's own or allogeneic body, modifying, expanding, and activating them in vitro, and then infusing them into the patient. ACT utilizes CAR-T cells, high-affinity TCR-modified T cells, or tumor-specific TILs to recognize tumor antigens. CAR-T/NK cell therapy has recently shown great promise in the treatment of hematologic malignancies, but still faces challenges in the treatment of solid tumors, primarily due to the dynamic and heterogeneous nature of tumor phenotypes. 3D organoid models provide a powerful tool for studying ACT immunotherapy by reproducing cellular heterogeneity and preserving cell–cell interactions [193]. However, the efficacy of chimeric antigen receptor (CAR) T cell therapy in immune-rejecting tumor microenvironments (TMEs) remains limited. To address this issue, Maus et al. developed T cells expressing anti-mesothelin CAR and combined them with secretory T cell engager molecules (TEAMs) [194]. Compared to single-target CAR-T cells, these engineered T cells showed better tumor targeting and cytotoxicity in patient-derived liver cancer immune organoids and pancreatic ductal adenocarcinoma (PDAC) mouse models with primary or metastatic liver lesions [194]. Similarly, Wu et al. constructed a new type of CAR-T cell targeting phosphatidylinositol proteoglycan-3 (GPC3) and verified its anti-tumor activity using a 3D microfluidic chip model [195]. Their results showed a synergistic effect between CAR-T cells and dendritic cells, suggesting the presence of strong immune cell crosstalk and enhanced tumor cell toxicity [195].

Currently, personalized medicine platforms using immune organoids have been applied to the treatment of various solid tumors. A group of biological samples obtained from patients are reconstructed into symbiotic immune-tumor organoids. For patients who cannot provide lymph nodes, peripheral blood mononuclear cells can be used instead of lymph nodes. The patient's own tumor is reconstructed in the form of immune organoids, and the tumor microenvironment is replicated by integrating tumor cells, related stroma, and tumor infiltrating leukocytes (TILs). These immune organoids showed good results in predicting individual chemotherapy responses in patients with colorectal cancer and gastroesophageal cancer, with a positive predictive value of 88% and a negative predictive value of 100%. In addition, the clinical responses of immune organoids from different patients to patient immunotherapy were basically the same. This platform provides new ideas for screening personalized immunotherapies [196].

Furthermore, TIL therapy has advantages due to its ability to simultaneously target multiple tumor antigens, and the development of organoid technology has also changed the way TIL treatment efficacy is evaluated. For example, bioprinting platforms can comprehensively assess TIL migration patterns and functional activities [197].

## Discussion: critical viewpoints of organoids

**Fig. 7:**
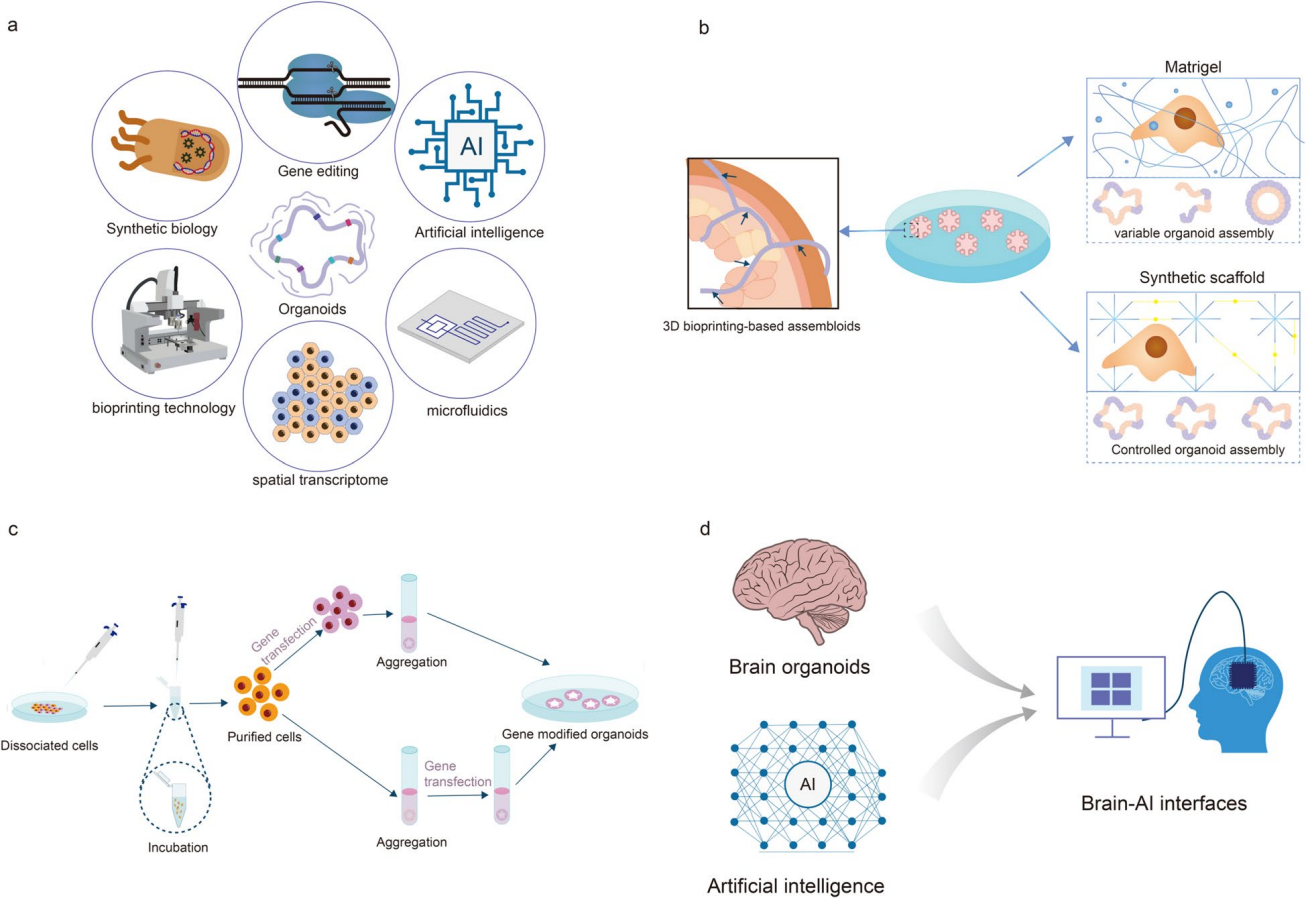
Outlook on organoid technology. **a** Organoids, as in vitro models with short modeling cycles and flexible modeling capabilities, can be combined with various technologies such as regenerative medicine, microfluidics, and spatial transcriptomics to form a composite culture platform. **b** Organoids can be combined with biomaterials or 3D bioprinting to replicate the spatial structure of complex tissues and solve the problem of chaotic organoid morphology. **c** Two culture strategies for constructing transgenic organoids: 1. Gene editing on a single cell followed by induction to self-assemble into organoids (high transfection efficiency, suitable for stem cells). 2. Constructing organoids first, then achieving gene modification through local transfection (suitable for mature cells). **d** Construction of organoid artificial intelligence (OI): Combining human brain and human organs with convolutional neural networks to construct a biological hybrid system with a human level cognitive model (HLM)

Organoid research has made significant contributions to disease modeling and drug screening, but some limitations impede their moving forward. Compared to animal models, the lack of a functional vascular system, nervous system, or immune system is a disadvantage of organoids, making them far inferior to in vivo models. In fact, in recent years, the development of organoids has further broadened the scalability of organoids as an in vitro culture model from multiple aspects, such as simplified construction systems [198], co-culture of multiple cell and organ sources [199], 3D biomaterial regenerative medicine [200], and the combination of spatial transcriptome and organoid technology [201]. This makes organoid culture a composite culture platform that can be combined with multiple technologies to conduct multi-omics mechanism research at a low cost (Fig. 7a). In the methodological research of organoid construction systems, in addition to the several more classic organoid construction systems introduced above, people have developed several new immune organoid construction methods. Although these methods offer promising improvements, their general applicability and scalability remain to be fully validated. To overcome the limitations of conventional immune organoid models, particularly the restricted functionality and structural diversity of co-cultures, Michael et al. transplanted human intestinal organoids (HIOs) into the renal capsule of humanized immune system mice. This strategy facilitated the infiltration of human immune cells into both the epithelial and lamina propria layers of the HIOs, effectively recapitulating aspects of fetal intestinal lymphoid follicle development. This in vivo model demonstrated dynamic immune-epithelial interactions within a tissue-like context [202]. Ankur et al. advanced the field by replacing conventional extracellular matrices such as Matrigel with synthetic hydrogels to create immune organoids capable of simulating the lymphatic microenvironment [203]. The researchers found that this system supported the proliferation, differentiation, and plasma cell generation of B cells derived from peripheral blood mononuclear cells (PBMCs), avoiding the need for tonsil-derived lymphocytes. This platform holds significant potential for translational research, including the modeling of personalized immune responses and evaluating immune reconstitution following B cell–targeted therapies [203]. Jeroen et al. integrated microfluidic platforms with Matrigel-based organoid culture to construct a microfluidic lymphatic vessel model [204]. This system was co-cultured with murine colon cancer organoids to investigate cell-induced lymphatic vessel formation and tumor cell migration. The microfluidic chip incorporated a compartmentalized extracellular matrix that maintained immune cell viability and functionality over extended culture periods [204]. Recently, researchers have even modified specific antibodies to target ECMβ1, a key protein in matrix gel that promotes organoid growth. By adding the single, well-defined molecule scTS2/16 exogenously, they have successfully constructed a highly efficient, chemically defined organoid culture system based entirely on type I collagen. This allows organoids to escape the "cage" of matrix gel culture, further reducing the cost of organoid culture and likely having great application in organoid research [205]. Collectively, these innovations represent meaningful advancements in immune organoid engineering. By refining structural integrity, improving immune cell integration, and extending culture life, these modified systems provide robust platforms for analyzing immune-tumor interactions, elucidating disease mechanisms, and identifying therapeutic targets with improved physiological relevance.

The construction of organoids is inseparable from a key element-biomaterials. They are not only the physical support for cell growth, but also the "scaffold" for regulating cell behavior and simulating tissue microenvironment. Selecting the right biomaterial is an important guarantee for achieving precise organoid structure and mature function. Although Matrigel is widely used, its composition is complex and there are large batch differences. Hydrogels, nanofibers, and ceramic materials are also gradually being widely used in organoid culture [206, 207]. In addition to the aforementioned biomaterials, 3D bioprinting technology can create a more physiological "growth environment" for organoids by precisely controlling the spatial distribution and arrangement of cells (Fig. 7b). Its key advantages lie in layer-by-layer printing according to pre-set patterns, replicating the spatial structure of complex tissues and addressing the issue of chaotic organoid morphology. Bioink design and parameter adjustment can mimic the mechanical and chemical microenvironment of real tissues, promoting intercellular interactions. A unified printing process reduces batch variability, making organoids more stable and reproducible. The integration of 3D bioprinting and organoids is moving the concept of "replicating human organs in vitro" from science fiction to reality. This technology not only provides more accurate models for basic research but also holds promise for breakthroughs in personalized medicine and organ transplantation in the future [208]. Despite the widespread application of biomaterials in organoids, the technology faces numerous challenges: the mechanisms of material-cell interactions need to be further clarified; multi-material and multi-scale printing technologies are still under development to create highly heterogeneous and functionally integrated organoids; and standardization and compliance are prerequisites for clinical translation, necessitating the promotion of GMP-grade production and quality control of biomaterials.

For organoids from multi-cell co-culture systems such as immune organoids, although immune organoids have made significant progress in tumor treatment, immune organoid models still face multiple technical challenges that restrict their wider application. One primary issue is the limited ability of current in vitro models to fully replicate the complex physiological environment of the in vivo immune system [209]. This restricts the accuracy of immune responses and cellular interactions within organoid cultures. To address this issue, it is essential to optimize co-culture systems by incorporating a broader range of immune cell types and refining culture conditions to preserve immune cell functionality. These improvements would help ensure that the physiological state of immune cells in vitro more closely resembles their original state [210]. Another major limitation is the relatively short lifespan of immune organoids derived from immune tissues, which typically survive for less than three weeks. This restricts the ability to study long-term immune responses or chronic disease mechanisms. Improving culture media formulations, modulating nutrient delivery, and incorporating supportive stromal elements may help extend organoid viability, expanding their experimental utility [65, 211].

More recently, the application of organoids in regenerative medicine has expanded beyond simple tissue regeneration or organ transplantation. The need for "living laboratories" to develop precision therapies has led to increasingly close collaborative research between organoids and gene editing [72]. The construction of gene-edited organoids requires overcoming two major technical hurdles: three-dimensional culture of organoids and targeted gene delivery. Currently, the mainstream approaches include two major types. Approach 1: Gene editing is performed on single cells, then their self-assembly into organoids is induced (high transfection efficiency, suitable for stem cells). Approach 2: Organoids are constructed first, then gene modification is achieved through local transfection (suitable for mature cells) (Fig. 7c) [72]. Currently, ectodermal organoids (brain organoids [212], skin organoids [213]), mesodermal organoids (kidney organoids [214], blood vessel organoids [215]), and endoderm organoids (intestinal organoids [216], pancreatic organoids [217]) have varying degrees of applications. However, the application of gene editing technologies, such as CRISPR, in organoids is still in its early stages, mainly concentrated on a few tissue types, and mostly on a small scale. To fully realize the potential of organoids in genome screening, it is necessary to address technical challenges, such as improving gRNA activity, reducing clonal heterogeneity, optimizing the timing of Cas9 induction, and developing more efficient screening strategies [218].

Beyond its traditional applications in drug screening, regenerative medicine, and mechanistic exploration, organoids are also being explored by scientists with the advancements in artificial intelligence (AI). Scientists are exploring the possibilities of combining AI and deep learning with organoids. The value of human brain organoids lies in their high biomimicry. They can self-organize into active neural networks with spontaneous oscillations and functional connections in vitro. Research shows that the neuronal ensembles within organoids exhibit synchronous activity, generate local field potentials (LFPs), and achieve phase-locking with neuronal spike activity. This indicates that organoids possess network-level dynamics similar to those of the living brain. Deeply integrating living brain organoids with AI technology can construct bio-hybrid systems with human-level cognitive models (HLMs)(Fig. 7d). This organoid intelligence (OI) can provide a novel, biologically based model for studying human cognition, thus opening new paths for bio-inspired computing [219]. However, current research on OI still faces many challenges. While current "bio-hybrid chip" robots integrate eye organoids, thalamic organoids, and motor neuron spheres, achieving mimicry of muscle contraction under light stimulation, demonstrating the integration potential of organoids in sensory-motor circuits, this remains a challenge. However, the neural systems of ocular organoids and motor neurons differ significantly from those of brain organoids, which are remarkably complex. Furthermore, maintaining the maturity and physiological realism of brain organoids through microenvironment optimization in the long term, and addressing the limitations of traditional planar electrode arrays in recording signals from freely floating organoids, are current challenges for OI models [219]. Developing brain-computer interfaces capable of effective interaction with three-dimensional organoid micro-tissues and creating new closed-loop AI-organoid co-adaptation systems are key technological bottlenecks that OI models need to overcome.

The success rate of culturing organoids from patient tissues is low, and efficiency needs to be improved by optimizing culture conditions and introducing regulatory factors. Also, it is difficult for organoids to fully replicate the complexity of tumor tissue, especially the diversity and temporal evolution of gene mutations. In addition, organoids lack key in vivo external stresses, such as hypoxia and biomechanical stimulation, which are crucial for cancer progression and treatment response. Simulating these external characteristics in organoid systems is still technically challenging and requires multidisciplinary collaboration and reference to innovations in the field of bioengineering.

In summary, organoid technology has made significant progress over the past two decades, boasting shorter construction cycles, higher success rates, and better performance in preserving individualized tissue characteristics of patients than some other in vitro cell culture models. This has brought trimendous new opportunities for biomedical research and clinical applications, demonstrating enormous potential in multiple fields, such as disease modeling, drug screening, and regenerative medicine. However, current research on organoids still faces substantial technical and ethical challenges before widespread clinical application. Due to their shorter construction cycles, organoids have broad application potential in both high-throughput drug screening and personalized medicine risk assessment, representing a paradigm shift in drug development. The integration of organoids with cutting-edge technologies such as synthetic biology, artificial intelligence, and gene editing heralds a transformative phase in biomedical innovation. This synergy is expected to unlock new therapeutic pathways, advance drug development, and provide profound new insights into complex diseases. The potential of organoids as rapid drug testing and functional experimental platforms, coupled with their compatibility with gene editing technologies, marks a quantum leap in medical research. As researchers and clinicians continue to harness the power of this technology, we anticipate a future filled with groundbreaking discoveries and innovative treatments that will ultimately reshape how we understand and treat human diseases.

## Concluding remarks

Organoid cultures, regardless of where it is derived from, offer a promising platform for immunological research with diverse applications. First, organoids allow for the reductive study of the complex and intimate interactions between epithelial and immune cells. The influence of the immune system on epithelial differentiation and function has been extensively studied in the intestine. It will be interesting to see whether similar principles apply to other organoid systems, such as the skin or lung, which also interact with commensal microorganisms and immune cells. Epithelial composition changes upon infection and in response to cytokine triggering. Thus, the potential bidirectional interactions between the immune system and the epithelium, such as those seen in the intestine, can be readily studied in vitro using organoids. Such studies will increase our understanding of the biology of complex inflammatory diseases, from IBD to multiple sclerosis, psoriasis, and asthma.

Organoids are also being used to study host-microbe interactions. A further step in this direction will be to add immune system components to infected organoids. A limited number of methods using this triple co-culture have been developed, primarily to simulate pathogenic infections with viruses or bacteria. Here, organoids are infected with the pathogen (virus or bacterium) before being co-cultured with immune cells. More recently, the commensal microbiota is increasingly shown playing a key role in maintaining mammalian homeostasis, which may be at least partially based on interactions with the epithelium. Epithelial organoid technology provides a unique opportunity to study all three components (epithelium, immune system, and commensal microbes) in a defined system. The interactions between these components play a crucial role in many human diseases, such as IBD and asthma. Therefore, a better understanding of these interactions and their consequences is of great interest. Future optimization of (triple) co-culture systems will be important for studying these interactions under both steady-state and pathological conditions and may lead to new therapeutic approaches.

Finally, tumor-derived organoids, which represent the transcriptional and mutational profiles of the original tumor, provide new and reliable model systems for the interaction of the immune system with tumor cells. Several tumor-derived organoid systems have been shown to faithfully recapitulate tumor responses to various therapies, including chemotherapeutic drugs and radiation therapy. This faithful recapitulation of in vitro tissue responses is also interesting in other pathological conditions and has been exemplified in the case of cystic fibrosis, where patient-derived organoids can be used to predict patient responses to treatment. The use of organoids as a platform for developing and testing personalized medicine approaches is attractive because they are essentially cultures of primary patient material. While this is currently possible for cystic fibrosis and cancer, it is also possible for inflammatory diseases that affect epithelial tissues. To this end, organoid-based bioassays need to be standardized and brought to clinical grade, and diagnostic tests that include organoids should be included in clinical trials.

As cancer immunotherapy assumes an increasingly important role in the clinic, several reports suggest that tumor-derived organoids can be used to model the effects of this novel therapeutic approach. However, while tumor-derived organoids show promise for future research, several important limitations should be considered. Organoids are typically derived from biopsies, which represent only a small portion of tumor tissue. Therefore, the complexity of the entire tumor is often underestimated by organoids. Furthermore, tumor-derived organoids are not exposed to external stresses that occur in situ, such as hypoxia or immune selection. Selection can influence the growth of tumor clones, resulting in situations where dominant clones in vitro are less dominant in situ, and vice versa. Importantly, these limitations become even more significant when considering hypermutated tumors, such as MMR-deficient CRC or NSCLC. More generally, organoid coculture conditions are often a compromise between optimal conditions for each included cell type. Since culture conditions can influence cell behavior in multiple ways, it is important to keep in mind when considering long-term coculture experiments. To this end, further optimizing coculture conditions, while keeping in mind factors such as culture medium composition and the type of ECM used, will be a key focus for researchers in the coming years. Despite these limitations, organoids currently provide the most accurate in vitro system for culturing human epithelial cells from virtually any organ and show great promise for future basic and translational research.

## Data Availability

Not applicable.

## Abbreviations

TME: Tumor microenvironment
2D: Two-dimensional
SCLC: Small-cell lung cancer
TMB: Tumor mutational burden
PDX: Patient-derived xenograft
ALI: Air–liquid interface
LBPA: Lysyl phosphatidic acid
CeD: Celiac disease
PDOs: Patient-derived organoids
MOS: Micro-organospheres
HIOs: Human intestinal organoids
PBMCs: Peripheral blood mononuclear cells
CAFs: Cancer-associated fibroblasts
NK: Natural killer
CRC: Colorectal cancer
ICB: Immune checkpoint blockade
ACT: Adoptive cell transfer
CAR: Chimeric antigen receptor
TEAM: T cell-engaging molecule
PDAC: Pancreatic ductal adenocarcinoma
GPC3: Glypican-3
TNBC: Triple-negative breast cancer
TBK1: TANK-binding kinase 1
